# Influence of Vanillin Acrylate-Based Resin Composition on Resin Photocuring Kinetics and Antimicrobial Properties of the Resulting Polymers

**DOI:** 10.3390/ma14030653

**Published:** 2021-01-31

**Authors:** Aukse Navaruckiene, Danguole Bridziuviene, Vita Raudoniene, Egidija Rainosalo, Jolita Ostrauskaite

**Affiliations:** 1 Department of Polymer Chemistry and Technology, Kaunas University of Technology, Radvilenu Rd. 19, LT-50254 Kaunas, Lithuania; aukse.navaruckiene@ktu.lt; 2 Biodeterioration Research Laboratory, Nature Research Center, Akademijos Str. 2, LT-08412 Vilnius, Lithuania; danguole.bridziuviene@gamtc.lt (D.B.); vita.raudoniene@gamtc.lt (V.R.); 3 Chemistry and Bioeconomy Team, Centria University of Applied Sciences, Talonpojankatu 2, FI-67100 Kokkola, Finland; egidija.rainosalo@centria.fi

**Keywords:** thermosets, bio-based polymers, photocuring, photoreometry, antimicrobial polymers, optical 3D printing

## Abstract

The investigation of the influence of vanillin acrylate-based resin composition on photocuring kinetics and antimicrobial properties of the resulting polymers was performed in order to find efficient photocurable systems for optical 3D printing of bio-based polymers with tunable rigidity, as well as with antibacterial and antifungal activity. Two vanillin derivatives, vanillin diacrylate and vanillin dimethacrylate, were tested in photocurable systems using phenyl bis(2,4,6-trimethylbenzoyl)phosphine oxide as a photoinitiator. The influence of vanillin acrylate monomer, amount of photoinitiator, presence and amount of dithiol, and presence of solvent on photocuring kinetics was investigated by real-time photoreometry. Polymers of different rigidity were obtained by changing the photocurable resin composition. The photocuring kinetics of the selected vanillin acrylate-based resins was comparable with that of commercial petroleum-based acrylate resins for optical 3D printing. Polymers based on both vanillin acrylates showed a significant antibacterial activity against *Escherichia coli* and *Staphylococcus aureus.* Vanillin diacrylate-based polymer films also demonstrated an antifungal activity in direct contact with *Aspergillus niger* and *Aspergillus terreus.* Vanillin diacrylate-based dual curing systems were selected as the most promising for optical 3D printing of bio-based polymers with antibacterial and antifungal activity.

## 1. Introduction

Recently, the search for bio-based photocurable resins for optical 3D printing has received considerable academic and industry attention due to the possibility of efficient development and production of sustainable products on-demand. Optical 3D printing is getting popular in the manufacturing companies since it is a cheap and fast way to produce new products in complex shapes [1]. It provides such benefits as lower material loss, reduced product weight, and the possibility to print spare parts without using fixtures or molds [2,3]. Optical 3D printing is used in various areas, such as dentistry [4], medicine [5], construction industry [6], etc. [7]. Increasing the popularity of optical 3D printing leads to the environmental problems, such as lack of bio-based materials with the same properties as petroleum-based materials [8] and recycling problems of the used products [9]. Additionally, nowadays, the antimicrobial activity of polymers is more important than ever. Food packaging for perishable products, such as meat and meat products [10], antimicrobial coatings for medical instruments, and implantable biomedical devices [11] are only a few examples of the possible application of such polymers.

One of the possible starting materials for the synthesis of bio-based polymers with an antimicrobial activity is vanillin [12]. Natural vanillin can be extracted from two different species of vanilla orchids: *Vanilla tahitensis* and *Vanilla planifolia* [13]. A cheaper way to obtain vanillin is the chemical modification of lignin [14]. Vanillin produced from lignin is considered as a natural vanillin [15] and is 250 times cheaper than synthetic vanillin [16]. Pure vanillin demonstrates impressive antibacterial activity against *Escherichia coli* and *Zygosaccharomyces rouxii* bacteria [13]. Some of the vanillin derivatives were also investigated for antibacterial and antifungal activity and were moderately active against them [17,18,19,20,21].

Photopolymerization is a valuable tool for the production of vanillin-based polymers. It can be used in a wide range of such areas as automotive, optical, and electronic equipment coatings [22], nanotechnology [23], stereolithography [24], medicine [25], etc. Photopolymerization is a fast polymerization process, which allows curing only the selected area of the product [22,26]. Vanillin acrylate, methacrylate, or acrylamide are the most common compounds for chain-growth polymerization [27]. Their polymerization is easily induced by UV light when the appropriate photoinitiator is used [28]. Vanillin-based photopolymers were successfully synthesized using acrylated epoxidized soybean oil as a comonomer [29].

Several photocuring techniques, free-radical photopolymerization, thiol-Michael photopolymerization, and dual curing combining both of them, were used in this study. Free-radical photopolymerization is relatively insensitive to impurities [30] and does not require the exclusion of moisture [31]. Heating is not required for this reaction, it can be run at room temperature or below [32] in solvent-free systems [33] with a spatial and temporal control of initiation [34] and it is a rapid process that occurs within a matter of minutes [35]. The main disadvantage of free-radical photopolymerization is the poor control of molecular weight and its distribution [36], as well as oxygen inhibition [37]. Thiol-Michael photopolymerization is a light activated reaction [38] which proceeds rapidly with no side products [39]. Its main advantages over free-radical photopolymerization are no inhibition by moisture and oxygen [40] and generation of homogenous materials [41]. Other advantages are high reaction rate [42], spatial and temporal control [43], and selective reactivity [44]. Dual curing is an effective tool to control the polymer network formation to obtain thermosets with desirable properties [45].

This work is a continuation of the previous studies [46,47] and focuses on the comparison of the influence of the resin composition on photocuring kinetics of free-radical, thiol-Michael, and dual curing systems with phenyl bis(2,4,6-trimethylbenzoyl) phosphine oxide (BAPO), as well as on antibacterial and antifungal properties of the resulting polymers. BAPO was selected for this study due to its advantage to release four radicals from a single monomer. Therefore, increasing the initiation performance [48] compared to ethyl (2,4,6-thimethylbenzoyl) phenyl phosphinate (TPOL) and diphenyl (2,4,6-trimethyl benzoyl) phosphine oxide (TPO) [46,47], which delivers two radicals [49]. Moreover, BAPO shows a more intensive absorbance, which indicates its higher photosensitivity and thus efficiency in comparison to TPOL and TPO [46,47]. BAPO is a type I photoinitiator which can participate in both thiol-Michael and radical photopolymerization reactions [50]. It does not require the addition of a co-initiator and generates radicals by a photocleavage process [51]. BAPO absorbs light at a relatively short wavelength, and as a result, polymers produced using BAPO have a very pale yellow color or no color at all [52]. These polymers demonstrate a high color stability and do not get yellow over time [53,54].

This work was performed in order to find efficient photocurable systems for optical 3D printing of bio-based polymers with tunable rigidity, as well as with antibacterial and antifungal activity.

## 2. Materials and Methods

Vanillin dimethacrylate (VDM) and vanillin diacrylate (VD) were purchased from Specific Polymers (Castries, France). 1,3-Benzenedithiol (BDT), phenyl bis(2,4,6-trimethylbenzoyl)phosphine oxide (BAPO), chitosan, and hydroxyethyl starch were purchased from Sigma-Aldrich (Darmstadt, Germany ). Dichloromethane (DCM) was purchased from Reachem Slovakia (Bratislava, Slovakia). All the materials were used as received. The FormLabs Clear FL6PCL02 resin was received from FormLabs (Somerville, MA, USA). The Autodesk Standard Clear Prototyping Resin (PR48) was received from Autodesk (Mill Valley, CA, USA).

**Figure 1:**
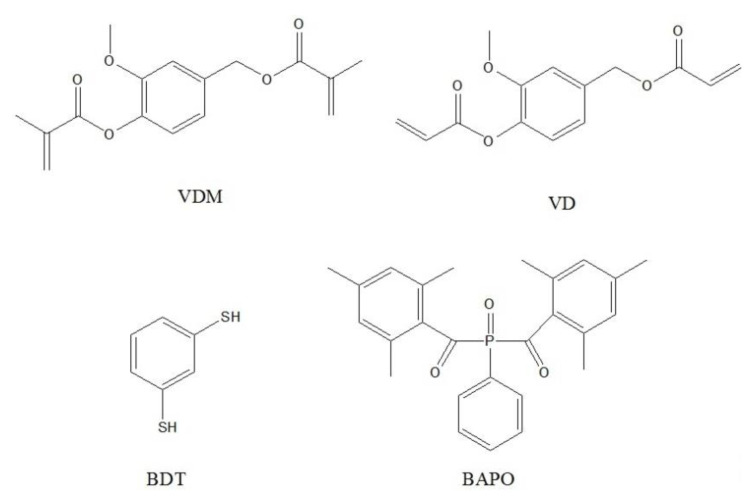
Structures of vanillin dimethacrylate (VDM), vanillin diacrylate (VD), 1,3-benzenedithiol (BDT), and phenylbis(2,4,6-trimethylbenzoyl)phosphine oxide (BAPO).

UV/Vis real-time photorheometry curing tests were performed with the resins containing 1 mol of vanillin derivative (VDM or VD), 0.5 or 1 mol of thiol (BDT) or without it, solvent DCM or without it, and 1, 3, or 5 mol% of photoinitiator (BAPO) (Figure 1) on a MCR302 rheometer (Anton Paar, Graz, Austria) equipped with the plate/plate measuring system. The Peltier-controlled temperature chamber with the glass plate (diameter 38 mm) and the top plate PP08 (diameter 8 mm) was used. The measuring gap was set to 0.1 mm. The samples were irradiated by UV/Vis light in a wavelength range of 250–450 nm through the glass plate of the temperature chamber using the UV/Vis spot curing system OmniCure S2000 (Lumen Dynamics Group Inc., Mississauga, ON, Canada). The shear mode with the frequency of 10 Hz and shear strain of 0.9% were used in all cases. The values of the storage modulus (*G*’) after 350 s of irradiation and the values of the gel point (*t*_gel_), defined as a crossover point of the storage modulus *G*’ and loss modulus *G*’’, were selected for the analysis during this study. The arithmetic average of each parameter of three measurements of each resin was calculated. The variation of the experimental results did not exceed 5% within the group.

Resin codes indicating the resin content were created as follows:

- 1 VDM or 1 VD shows that 1 mol of vanillin derivative, vanillin dimethacrylate (VDM), or vanillin diacrylate (VD) was used;
- 1 BDT or 0.5 BDT shows the presence and amount (1 mol or 0.5 mol) of 1,3-benzenedithiol;
- the number before BAPO indicates the concentration of the photoinitiator (1 BAPO means that 1 mol% of BAPO, 3BAPO—3 mol% of BAPO, and 5 BAPO—5 mol% of BAPO were used);
- DCM indicates the presence of dichloromethane in the resin composition.

For example, **1VDM/1BDT/1BAPO/DCM** is a resin composed of 1 mol of vanillin dimethacrylate, 1 mol of 1,3-benzenedithiol, 1 mol% of bis(2,4,6-trimethylbenzoyl)phosphine oxide, and a minimal amount of dichloromethane is needed to dissolve the solid components (0.25 g of DCM for 1 g of vanillin acrylate).

The vanillin-based polymer films were prepared by mixing all the materials with a magnetic stirrer at room temperature (25 °C) until the homogenous phase was reached and then poured into a Teflon mold and cured for 1–2 min under an UV lamp (Helios Italquartz, model GR.E 500 W, Milan, Italy) with UV/Vis light at an intensity of 310 mW/cm^2^. Photocross-linked polymer films were not washed after the UV curing. The films of chitosan and hydroxyethyl starch were prepared by casting from aqueous solutions (2% of chitosan and 9% of hydroxyethyl starch) of the commercial solid powders and drying at room temperature (25 °C).

The yield of insoluble fraction was determined by Soxhlet extraction. Polymer samples of 0.5 g were extracted with acetone for 24 h. After the extraction, the insoluble fractions were dried under a vacuum until no changes of the weight were observed. The yield of the insoluble fraction was calculated as a difference of the weight before extraction and after extraction and drying. The arithmetic average of the yield of insoluble fraction of the three film samples of each polymer was calculated. The variation of the experimental results did not exceed 5% within the group.

The swelling value of the cross-linked polymer specimens was obtained by measuring the volume of specimens swollen in acetone and toluene at 25 °C. The polymer film specimens of 10 (±0.1) mm length, 3 (±0.00) mm width, and 1.5 (±0.1) mm thickness were used. The initial volume of the polymer specimen was measured before placing it into the measuring container. After the solvent was poured into the measuring container, the change of the volume of the swelling agent was measured every 5 min until no change was obtained.

(1) $$\alpha \;=\frac{V\;-\;V_{0\;}}{V_{0}}\;\cdot 100$$

where *α* is a swelling value (%); *V* is a volume of the swollen specimen (mL); and *V*_0_ is an initial volume of the specimen (mL). The arithmetic average of the swelling value of the three film samples of each polymer was calculated. The variation of the experimental results did not exceed 5% within the group. No change of the volume was observed after 90 min for all the polymer samples. This value was taken for the comparison of swelling values of different polymers.

The study of the antimicrobial (antibacterial and antifungal) activity of polymers was performed in two ways: By the contact of polymer film specimens with a microbial growing culture on a solid medium and by the direct contact with microbial spores. The test microorganisms were Gram-negative bacterium *Escherichia coli* ATCC 25,922 (*E. coli*) and Gram-positive bacterium *Staphylococcus aureus* ATCC 29,213 (*S. aureus*), which were procured from the American Type Culture Collection (Manassas, VA, USA)., as well as fungal strains: *Aspergillus niger* 1089-13 (*A. niger*) and *Aspergillus terreus* 1084-10 (*A. terreus*) which were taken from the collection of Biodeterioration Research Laboratory, Nature Research Center (Vilnius, Lithuania).

In the first way performed according to ISO 846:1998 [55], Petri dishes with a Mueller Hinton Agar (MHA, Liofilmchem, Roseto degli Abruzzi, Italy) medium were inoculated with test bacterium *E. coli* or *S. aureus* and dishes with a Malt Extract Agar (MEA, Liofilmchem, Italy) medium were inoculated with *A. niger* or *A. terreus*. The test specimens of vanillin-based polymers and chitosan in dimensions of 10 mm × 10 mm × 0.4 mm and hydroxyethyl starch in dimensions of 20 mm × 20 mm × 0.4 mm were placed on the medium in the center of the dishes. Chitosan and hydroxyethyl starch were used as reference polymers. The films of chitosan and hydroxyethyl starch were prepared by casting from aqueous solutions. The dishes with bacteria were incubated for 48 h at 35 ± 2 °C and the dishes with fungi were incubated for 5 days at 26 ± 2 °C. After incubation, the antimicrobial activity was evaluated by the growth inhibition zones (mm) of microorganisms that have shown up around the specimens. Each specimen was tested in triplicate experiments. The mean value of the zones and mean standard deviation was calculated.

The second way of the polymer antimicrobial activity estimation was performed by a direct polymer film specimen inoculation with the suspension of microbial spores. The concentration of the inoculum of bacterium suspension was assessed with a spectrophotometer (Evolution 60S, Thermo Fisher Scientific, Waltham, MA, USA) at 600 nm and fungal suspension at 530 nm, then corrected by seeding the bacterium suspension on a Mueller Hinton Agar (MHA, Liofilmchem, Italy) and the fungal suspension on a Malt Extract Agar (MEA, Liofilmchem, Italy). The final inoculum concentrations were 6 × 10^5^ for *E. coli*, 7 × 10^5^ for *S. aureus*, 2.2 × 10^6^ for *A. niger*, and 2 × 10^6^ colony forming units/mL (CFU/mL) for *A. terreus*. The testing specimens of vanillin-based polymers and chitosan in dimensions of 10 mm × 10 mm × 0.4 mm, and the testing specimens of hydroxyethyl starch in dimensions of 20 mm × 20 mm × 0.4 mm were placed into 50 mm diameter sterile Petri dishes, inoculated with 10 µl of prepared bacterial or fungal suspension and incubated in humid chambers with bacteria at 35 ± 2 °C and with fungi at 26 ± 2 °C. Each specimen was tested in triplicate experiments. After 24 h, the specimens were washed with 2 mL of saline (0.9%) and serial dilutions of culture suspensions were sown on MHA for bacteria and on MEA for fungi in Petri dishes. The dishes with bacteria were incubated for 48 h at 35 ± 2 °C and the dishes with fungi were incubated for 5 days at 26 ± 2 °C. After incubation, colony numbers were counted and the percent reduction was calculated according to the formula: (*a* − *b*)/*a* × 100%, where *a* is the concentration of the colony forming units (CFU/mL) in inoculum suspension; *b* is a mean of the recovered spores (CFU/mL) on specimens from triplicate experiments after incubation. The log reduction of viable spores was calculated according to the formula: log(*a*)−log(*b*), where *a* is the concentration of the colony forming units (CFU/mL) in the inoculum suspension; *b* is a mean of the recovered spores (CFU/mL) on specimens from triplicate experiments after incubation.

Statistical Analysis. The collected data were statistically analyzed using ANOVA for the Microsoft Excel programme. All the experiments were performed three times and the results were assumed as the average values ± standard deviation. The estimated *p*-value was below 0.05 within the groups.

## 3. Results and Discussion

### 3.1. Influence of Resin Composition on Photocuring Kinetics

The photocuring kinetics of vanillin acrylate-based photocurable resins of different compositions was investigated by real-time photoreometry and compared. The values of the storage modulus (*G*’) and the gel point (*t*_gel_) were analyzed during this study. The trends of loss modules are the same as those of the storage modules during irradiation of all vanillin-based resins, thus, only storage modules will be analyzed in this work.

The gel point is a point at which a high-viscosity Newtonian fluid turns into a solid elastic material [52]. It characterizes the formation of the polymer network. The storage modulus is a measure of the deformation energy stored by the sample during the shear process and representing the elastic behavior of the material [56]. It characterizes the rigidity of the resulting polymers.

#### 3.1.1. Influence of Photoinitiator Concentration

**Figure 2:**
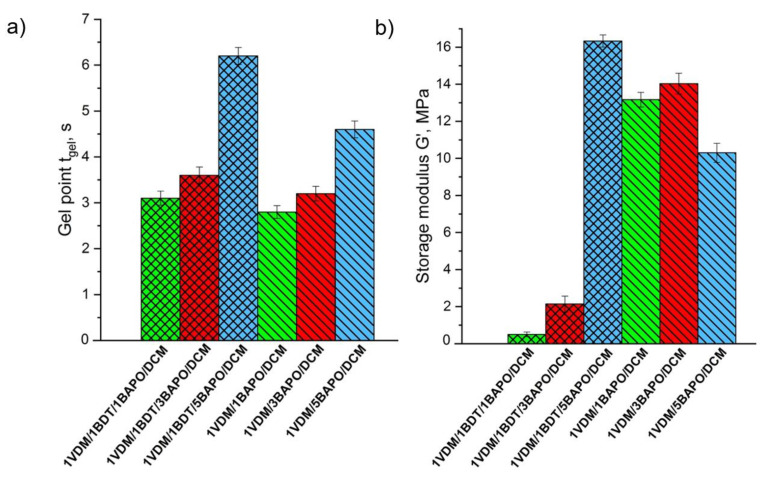
Gel point *t*_gel_ (**a**) and storage modulus *G*’ (**b**) of the VDM-based resins. Green columns—1 mol% of BAPO; red columns—3 mol% of BAPO; blue columns—5 mol% of BAPO; leaning lines from bottom to top—presence of 1,3-benzenedithiol (BDT); leaning lines from top to bottom—presence of dichloromethane (DCM).

VDM-based resins prepared with or without BDT showed a clear dependence of an increase in the *t*_gel_ value with the increasing BAPO concentration (Figure 2a). BAPO is a highly reactive photoinitiator and its very low concentration is sufficient to initiate polymerization effectively. In VDM-based resins, the lowest value of *t*_gel_ was determined when 1 mol% of BAPO was used. However, at higher photoinitiator concentrations, the *t*_gel_ values increased. It could be due to the generation of the higher concentration of free radicals at the surface, which block the sufficient energy from penetrating, leading to the reduced rate of polymerization [57]. The *t*_gel_ values of the VDM-based resins with or without BDT and 1–3 mol% of BAPO are very similar. However, a significant reduction of the *G*’ values was obtained when BDT was used, showing the formation of linear polymer chains with flexible thioether linkages [52] in these cases (**1VDM/1BDT/1BAPO/DCM**, **1VDM/1BDT/3BAPO/DCM**) (Figure 2b). The higher the *G*’ value and rigidity of the resulted VDM-based polymer with BDT (**VDM/1BDT/5BAPO/DCM**) was obtained probably due to the increase of termination reactions of macroradicals with free radicals which usually occur at the high photoinitiator concentration [48,58], and thus the shorter polymer chains leading to the less rigid polymer were formed in this case. The *G*’ values of pure VDM resins increased with an increase in the BAPO concentration (**VDM/1BAPO/DCM, VDM/3BAPO/DCM**). However, the *G*’ value of the resin with 5 mol% of BAPO (**VDM/5BAPO/DCM**) was lower due to the effect of termination reactions.

**Figure 3:**
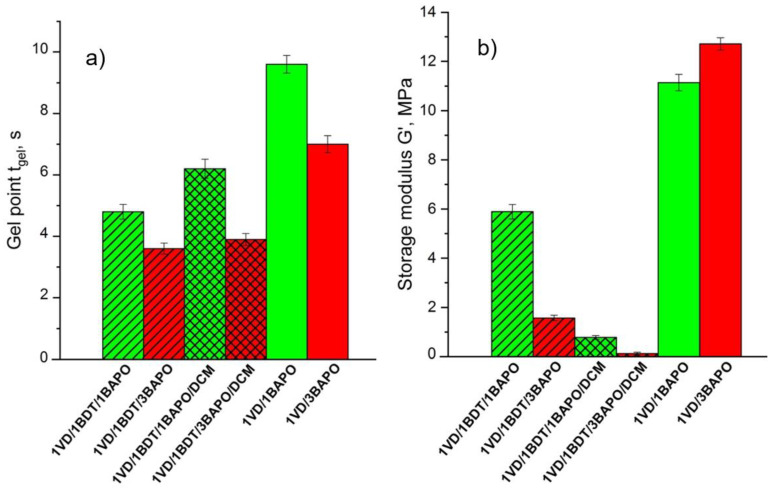
Gel point *t*_gel_ (**a**) and storage modulus *G*’ (**b**) of the VD-based resins. Green columns—1 mol% of BAPO; red columns—3 mol% of BAPO; leaning lines from bottom to top—presence of BDT; leaning lines from top to bottom—presence of DCM.

Acrylates are more reactive than methacrylates, as secondary acrylate radicals are very unstable in comparison to highly stable tertiary methacrylate radicals [59]. Due to this reason, the lower *t*_gel_ values and thus the higher polymerization rate of VD-based resins were obtained with an increase in the BAPO concentration. The comparison of the *t*_gel_ and *G*’ values of VD-based resins prepared with 1 and 3 mol% of BAPO is presented in Figure 3. The lower *t*_gel_ values and the higher photocuring rate were demonstrated by VD-based resins when 3 mol% rather than 1 mol% of BAPO were used. The *t*_gel_ values of the resins prepared with thiol and the pure VD-based resin without solvent, **1VD/1BDT/3BAPO**, **1VD/1BDT/3BAPO/DCM**, and **1VD/3BAPO** were 3.6, 3.9, and 7.0 s, respectively (Figure 3a). VD-based resins prepared with BDT and with or without DCM resulted in more rigid polymers when 1 mol% of BAPO was used (**1VD/1BDT/1BAPO**, **1VD/1BDT/1BAPO/DCM**), while the pure VD-based resin resulted in a more rigid polymer when 3 mol% of BAPO were used (**1VD/3BAPO**) (Figure 3b). It was due to the different mechanisms of photopolymerization. The higher amount of photoinitiator in the thiol-Michael photopolymerization (when BDT was used in the resins) resulted in a higher concentration of thiolate anions and the formation of a higher amount of linear polymer chains with flexible thioether linkages [60,61] and thus, the lower rigidity of polymers (Figure 3b).

**Figure 4:**
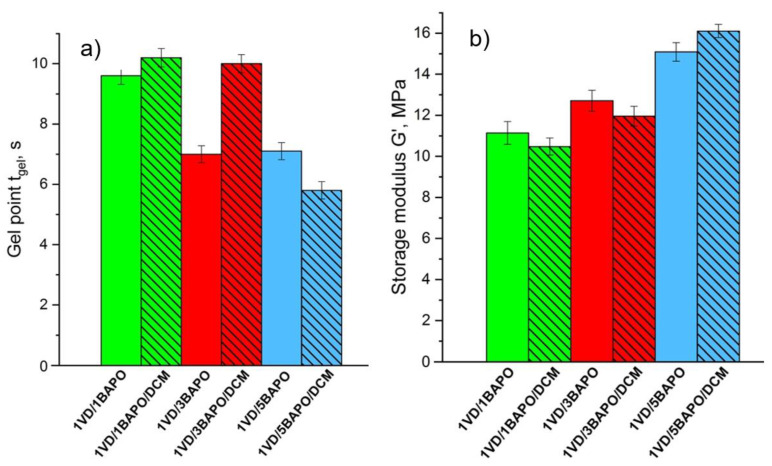
Gel point *t*_gel_ (**a**) and storage modulus *G*’ (**b**) of the VD-based resins. Green columns—1 mol% of BAPO; red columns—3 mol% of BAPO; blue columns—5 mol% of BAPO; leaning lines from top to bottom—presence of DCM.

Photocuring of resin **1VD/5BAPO/DCM** with 5 mol% of BAPO was the fastest (gel point 5.8 s) compared with resin **1VD/1BAPO/DCM** containing 1 mol% of BAPO (gel point 10.2 s) and resin **1VD/3BAPO/DCM** containing 3 mol% of BAPO (gel point 10.0 s) (Figure 4a). The solvent created a more homogenous phase for resin **1VD/5BAPO/DCM** and that led to a better dissolution of BAPO, faster photocuring, and the formation of a more rigid polymer. In pure VD-based systems with and without DCM, the rigidity increased gradually with the increase of the photoinitiator concentration since a high amount of molecules was available for the generation of free radicals. The highest rigidity was shown by polymers with 5 mol% of BAPO, while the lowest was shown with 1 mol% of BAPO (Figure 4b), as the higher concentration of reactive species resulted in a higher rigidity of polymers [62].

For a further investigation, 3 mol% of BAPO was chosen as it resulted in faster or only a slightly slower photocuring than that with 1 mol% of BAPO. However, 5 mol% of BAPO could not be used in further studies due to the left undissolved particles of BAPO in polymers **1VD/1BDT/5BAPO** and **1VD/5BAPO,** which were prepared without DCM.

#### 3.1.2. Influence of Dichloromethane

VD-based resins were selected to characterize the influence of DCM to the photocuring rate and rigidity of the obtained polymers since only VD-based resins could be prepared without a solvent, as VD is in a liquid state at room temperature while VDM is a solid material.

**Figure 5:**
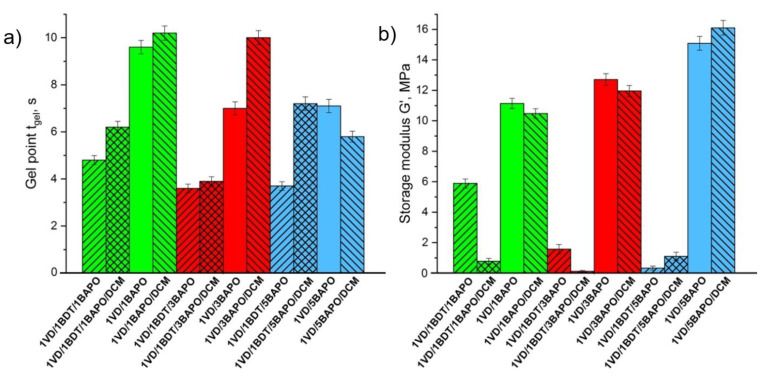
Gel point *t*_gel_ (**a**) and storage modulus *G*’ (**b**) of the VD-based resins. Green columns—1 mol% of BAPO; red columns—3 mol% of BAPO; blue columns—5 mol% of BAPO; leaning lines from bottom to top—presence of BDT; leaning lines from top to bottom—presence of DCM.

In most cases, DCM slowed down the photocuring process and less rigid polymers were obtained (Figure 5). These results were characteristic for all resins with 1 and 3 mol% of BAPO. This can be explained by the DCM action as a chain transfer agent slowing the photocuring process [58]. For example, the resin without solvent **1VD/3BAPO** reached *t*_gel_ after 7 s and *G*’ of 12.7 MPa after 350 s, while the same resin with solvent **1VD/3BAPO/DCM** reached *t*_gel_ only after 10 s and *G*’ of 12.0 MPa after 350 s.

The same results were obtained for the VD-based resins with 5 mol% of BAPO, resin **1VD/1BDT/5BAPO** photocured faster than **1VD/1BDT/5BAPO/DCM**. The addition of DCM lowered the concentration of monomers in the resin and led to more chain transfer reactions [50], which resulted in a high *t*_gel_ value and lower G’ of **1VD/1BDT/5BAPO/DCM**. Different results were obtained for the pure VD-based resins with 5 mol% of BAPO, **1VD/5BAPO** and **1VD/5BAPO/DCM,** which undergo a free radical photopolymerization mechanism. The concentration of BAPO was too high in resin **1VD/5BAPO** and small undissolved particles of BAPO were still visible in the polymer after photocuring. In this case, a more rigid polymer was obtained when DCM was used, as a solvent homogenized resin by dissolving the high amount of photoinitiator particles. Therefore, resin **1VD/5BAPO/DCM** resulted in a higher rigidity of the obtained polymer and faster curing (*t*_gel_ = 5.8 s, *G*’ = 16.1 MPa) than that of **1VD/5BAPO** (*t*_gel_ = 7.1 s, *G*’ = 15.1 MPa).

#### 3.1.3. Influence of Vanillin Derivative

**Figure 6:**
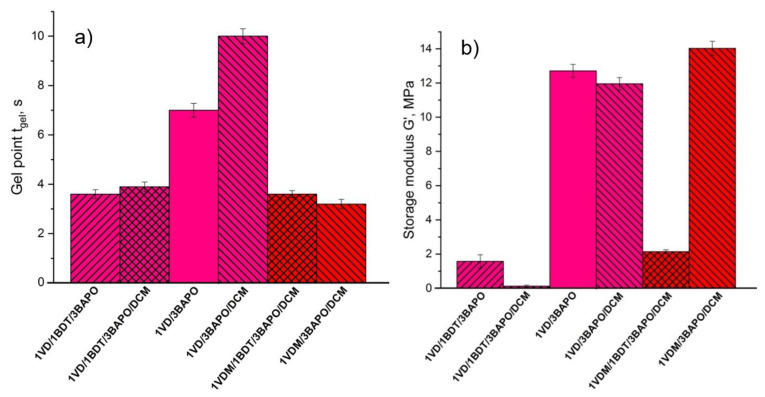
Gel point *t*_gel_ (**a**) and storage modulus *G*’ (**b**) of the VD and VDM-based resins with 3 mol% of BAPO. Pink columns—VD-based resin; red columns—VDM-based resin; leaning lines from bottom to top—presence of 1.3 BDT; leaning lines from top to bottom—presence of DCM.

**Figure 7:**
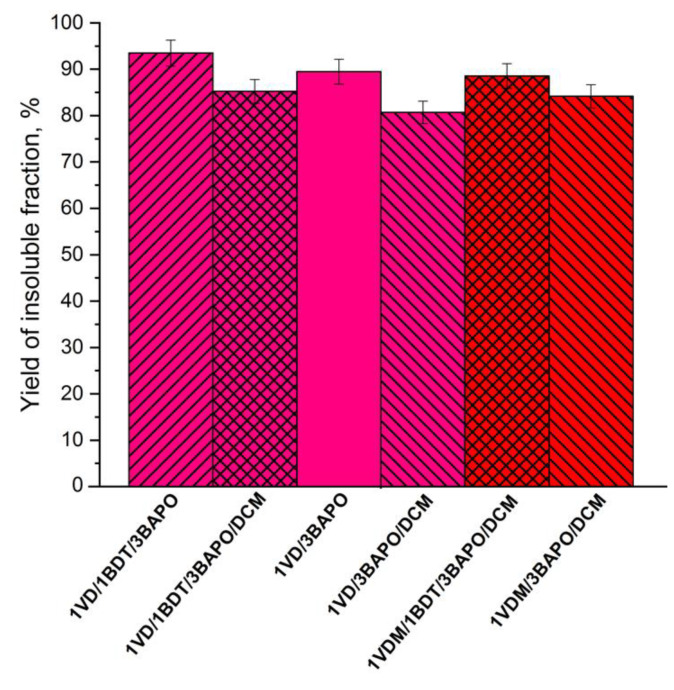
The yield of insoluble fraction of the VD and VDM-based polymers with 3 mol% of BAPO after 24 h extraction in acetone. Pink columns—VD-based polymer; red columns—VDM-based polymer; leaning lines from bottom to top—presence of 1.3 BDT; leaning lines from top to bottom—presence of DCM.

**Figure 8:**
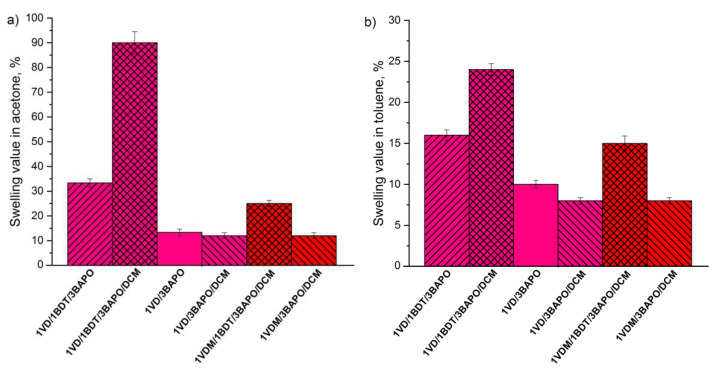
The swelling values of polymers in acetone (**a**) and in toluene (**b**) after 90 min of the VD and VDM-based polymers with 3 mol% of BAPO. Pink columns—VD-based polymer; red columns—VDM-based polymer; leaning lines from bottom to top—presence of 1.3 BDT; leaning lines from top to bottom—presence of DCM.

A series of resins with different vanillin derivatives (VDM or VD), DCM, and 3 mol% of BAPO were selected to determine the influence of vanillin acrylate monomer to photocuring kinetics (Figure 6). In all these cases, more rigid polymers were formed from VDM-based resins compared to VD-based resins due to the higher stability of methacrylate radicals, which slow down the photopolymerization process and form a more uniform polymeric network [59]. The VDM-based resin without thiol **1VDM/3BAPO/DCM** reached *G*’ of 14.00 MPa after 350 s, while *G*’ of the VD-based resin without thiol **1VD/3BAPO/DCM** was 12.00 MPa after 350 s. As for the reaction rate, the VDM-based resin **1VDM/3BAPO/DCM** reached the gel point faster than the VD-based resin **1VD/3BAPO/DCM** (3.2 and 10.0 s, respectively). VD-based resins are supposed to polymerize faster than VDM-based ones, but in this case, the reaction was slowed down by the color of the VD. VDM is a white color powder which results in a colorless resin when dissolved in DCM. VD is a dark yellow liquid which results in a yellow resin. The darker color makes it harder for the light to reach deeper layers of the resin and polymerize it [63]. This was confirmed by the swelling values and the yield of insoluble fraction. VDM-based polymers demonstrated a higher yield of insoluble fraction and lower swelling values in both tested solvents. The yield of insoluble fraction of the VDM-based polymer without thiol **1VDM/3BAPO/DCM** was 84.14% when the VD-based polymer without thiol **1VD/3BAPO/DCM** was 80.71% (Figure 7). The swelling values observed after 90 min in acetone and toluene correlates to the yield of insoluble fraction. The VDM-based polymer without thiol **1VDM/3BAPO/DCM** reached the swelling value of 12% in acetone and 8% in toluene, while the VD-based polymer without thiol **1VD/3BAPO/DCM** reached the swelling value of 20% in acetone and 10% in toluene (Figure 8). Resins, prepared with thiol demonstrated similar results. The VDM-based resin with thiol **1VDM/1BDT/3BAPO/DCM** reached *G*’ of 2.14 MPa after 350 s, while *G*’ of the VD-based resin without thiol **1VD/1BDT/3BAPO/DCM** was 0.12 MPa after 350 s. Both resins reached *t*_gel_ at a similar time, but the VDM-based resin reached it slightly faster (3.6 s) than the VD-based resin (3.9 s), as a result of the darker color of the VD-based resin [63]. VDM-based polymers with thiols also demonstrated a higher yield of insoluble fraction and lower swelling values in both tested solvents. The yield of insoluble fraction of the VDM-based polymer with thiol **1VDM/1BDT/3BAPO/DCM** was 88.54% when the VD-based polymer with thiol **1VD/1BDT/3BAPO/DCM** was 85.22% (Figure 7). The swelling values observed after 90 min in acetone and toluene correlates to the yield of insoluble fraction. The VDM-based polymer with thiol **1VDM/1BDT/3BAPO/DCM** reached the swelling value of 25% in acetone and 15% in toluene, while the VD-based polymer with thiol **1VD/1BDT/3BAPO/DCM** reached the swelling value of 90% in acetone and 24% in toluene (Figure 8). Higher swelling values were obtained in acetone as vanillin acrylates are soluble in acetone and insoluble in toluene. The poor interaction of vanillin acrylate-based polymer chains with toluene was the reason for the worse swelling than in acetone. The higher swelling values and lower yields of insoluble fractions showed that longer chains between cross-linking points were formed.

#### 3.1.4. Influence of Thiol Addition and Its Amount

The reaction mechanism had a significant influence on the photocuring kinetics of VDM-based and VD-based resins. BAPO is a universal photoinitiator, which can initiate both free-radical and thiol-Michael photopolymerizations. The free-radical photopolymerization occurred when only vanillin acrylate was used as a monomer. The thiol-Michael photopolymerization occurred in vanillin acrylate-based resins containing BDT, when the ratio of the acrylate and thiol groups was 1:1 and 1:0.5. Dual curing, combining free-radical and thiol-Michael mechanisms, occurred when a lower amount than the stoichiometric of thiol was used (acrylate: thiol, 1:0.5). The polymers with the higher amount of thioether bonds are formed from the resins with the stoichiometric ratio of acrylate and thiol monomers (acrylate: thiol, 1:1), while the polymers with the higher amount of carbon-carbon bonds are formed from the resins with the lower than stoichiometric amount of thiol (acrylate: thiol, 1:0.5). The incorporation of thiol fragments into polymer chains leads to the significant changes of polymer mechanical properties [52]. How this has affected the rigidity of vanillin-based polymers will be described below. Nevertheless, both hard and rigid as well as soft and flexible polymers are desirable for optical 3D printing of thermoset products for particular applications.

**Figure 9:**
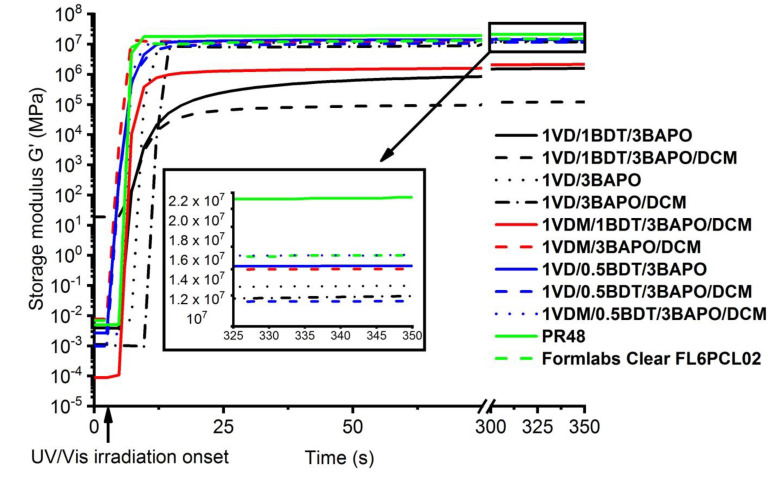
Dependencies of storage modulus *G*’ of the VD and VDM-based resins, containing 3 mol% of BAPO and commercial resins PR48 and Formlabs Clear FL6PCL02 on the irradiation time.

The resins with 3 mol% of BAPO were selected to investigate the influence of thiol addition and its amount on vanillin acrylate-based resin photocuring kinetics. In vanillin acrylate-based resins, different results of the reaction rate (gel point) were observed using free radical and thiol-Michael mechanisms, as it could be expected. For example, the *G*’ curves of VD-based resins prepared with thiol and with or without the solvent, **1VD/1BDT/3BAPO** and **1VD/1BDT/3BAPO/DCM**, and VD-based resins prepared without thiol and with or without the solvent, **1VD/3BAPO** and **1VD/3BAPO/DCM**, are presented in Figure 9. In all these cases, the reaction was faster when resins were prepared with the stoichiometric amount of thiol (acrylate: thiol,1:1), due to the high reactivity of the thiol radicals formed in the initiation stage of polymerization [52]. Moreover, BDT had a significant influence on the rigidity of the polymers. In all cases, polymers were less rigid when the stoichiometric amount of BDT (acrylate: thiol,1:1) was used in the resins. It was due to the formation of many different polymer chains of different lengths with flexible thioether linkages [48]. For example, the VD-based resin with thiol **1VD/1BDT/3BAPO** reached the gel point after 3.6 s and its *G*’ was only 1.7 MPa after 350 s, while the VD-based resin without thiol **1VD/3BAPO** reached the gel point only after 7.0 s, but the obtained *G*’ was 12.7 MPa after 350 s. As for the resins based on VDM, the reaction was slightly slower, when thiols were used. VDM has a lower reactivity than the VD due to the higher stability of methylacrylate radicals. This stability slows down the thiol-Michael photopolymerization [64]. The VDM-based resin with thiol and solvent **1VDM/1BDT/3BAPO/DCM** reached the gel point after 3.6 s and the VDM-based resin with the solvent and without thiol **1VDM/3BAPO/DCM** reached the gel point after 3.2 s.

The analogous series of the resins with the lower than the stoichiometric amount of thiol (acrylate: thiol, 1:0.5) and 3 mol% of BAPO were prepared by combining the free-radical and thiol-Michael photopolymerization in order to get the dual curing systems. Resins **1VD/0.5BDT/3BAPO**, **1VD/0.5BDT/3BAPO/DCM**, and **1VDM/0.5BDT/3BAPO/DCM** resulted in a 2 s slower photocuring (*t*_gel =_ 5.3, 5.8, and 5.3 s, respectively) than the resins polymerized only by the thiol-Michael reaction mechanism (acrylate: thiol, 1:1). However, all the polymers prepared with a 0.5 mole of BDT demonstrated a higher rigidity than the polymers prepared with 1 mole of BDT. The VD-based resin with a 0.5 mole of thiol and 3 mol% BAPO (**1VD/0.5BDT/3BAPO**) reached *G*’ of 14.3 MPa after 350 s, which was higher than that of the pure VD-based polymer synthesized with 3 mol% of BAPO (**1VD/3BAPO**, *G*’ = 12,7 MPa, after 350 s). The analogous polymer prepared with solvent (**1VD/0.5BDT/3BAPO/DCM**) was slightly less rigid and reached *G*’ of 11.6 MPa after 350 s. The addition of solvent into the resin also slightly slowed down the photocuring process and the rigidity of polymers due to the chain transfer reactions [61]. Similar results were observed for the VDM-based resin with the lower than stoichiometric amount of thiol (acrylate: thiol, 1:0.5), 3 mol% of BAPO, and DCM (**1VDM/0.5BDT/3BAPO/DCM**). The resulted polymer reached *G*’ of 15.2 MPa after 350 s, which was higher than that of the VDM homopolymer synthesized with 3 mol% of BAPO and DCM (**1VDM/3BAPO/DCM**, *G*’ = 14.0 MPa, after 350 s). The higher rigidity of polymers, prepared with a 0.5 mole of thiol is the result of the dual curing process. During this process, the macromolecular chains of acrylate homopolymer and copolymer interpenetrate. That results in a higher density and thus the higher rigidity of polymers [45]. On the other hand, the free-radical photopolymerization is slower than the thiol-Michael photopolymerization and this combination of the two reaction mechanisms (when acrylate: thiol, 1:0.5) results in a slower photocuring process than the thiol-Michael photopolymerization (when acrylate: thiol, 1:1) [61].

The viscosity of the resins depended on their composition. The higher amount of BDT was added to the resin, the lower viscosity of the resin was determined. The most viscous resin was **1VD/3BAPO** without BDT. Its viscosity was 75,024 ± 137 mPa·s. The viscosity of the resin with 0.5 mol of BDT (**1VD/0.5BDT/3BAPO**) was 3007 ± 42 mPa·s, and that with 1 mol of BDT (**1VD/1BDT/3BAPO**) was 468 ± 15 mPa·s. No change in the resin viscosity was observed and no difference was observed between the UV curing kinetics curves of freshly prepared vanillin-based resins containing BDT or without it and those kept in the dark for several days.

A series of the resins with 3 mol% of BAPO were compared with commercial petroleum-derived acrylate resins, Formlabs Clear FL6PCL02 and PR48 (Figure 9). Both commercial resins demonstrated a similar photocuring rate (both *t*_gel_ = 6 s). Most of the VD-based and VDM-based resins demonstrated an even higher photocuring rate than commercial resins, for example, **1VD/1BDT/3BAPO** (*t*_gel_ = 3.6 s), **1VD/1BDT/3BAPO/DCM** (*t*_gel_ = 3.9 s), **1VDM/1BDT/3BAPO/DCM** (*t*_gel_ = 3.6 s), **1VDM/3BAPO/DCM** (*t*_gel_ = 3.2 s), **1VD/0.5BDT/3BAPO** (*t*_gel_ = 5.3 s), **1VD/0.5BDT/3BAPO/DCM** (*t*_gel_ = 5.8 s), and **1VDM/0.5BDT/3BAPO/DCM** (*t*_gel_ = 5.3 s). Only pure VD-based resins with and without the solvent, **1VD/3BAPO** (*t*_gel_ = 7.0 s) and **1VD/3BAPO/DCM** (*t*_gel_ = 10.0 s), demonstrated the lower photocuring rate than that of commercial resins due to the lower rate of free radical photopolymerization. As for rigidity, VD-based and VDM-based resins prepared with or without the solvent and without thiol, **1VD/3BAPO** (*G*’ = 12.70 MPa), **1VD/3BAPO/DCM** (*G*’ = 12.00 MPa), and the pure VDM-based resin with the solvent **1VDM/3BAPO/DCM** (*G*’ = 14.00 MPa) showed a similar rigidity as Formlabs Clear FL6PCL02 (*G*’ = 15.20 MPa), but a lower rigidity than PR48 (*G*’ = 21.40 MPa). Dual-cured polymers demonstrated a similar rigidity, **1VD/0.5BDT/3BAPO** (*G*’ = 14.23 MPa), **1VD/0.5BDT/3BAPO/DCM** (*G*’ = 11.60 MPa), and **1VDM/0.5BDT/3BAPO/DCM** (*G*’ = 15.20 MPa), and the VDM-based resin **1VDM/0.5BDT/3BAPO/DCM** demonstrated even the same rigidity as Formlabs Clear FL6PCL02 (*G*’ = 15.20 MPa), but a lower rigidity than PR48 (*G*’ = 21.40 MPa). However, the VDM homopolymer **1VDM/3BAPO/DCM** was very brittle, so vanillin diacrylate-based double-curing systems **1VD/0.5BDT/3BAPO** and **1VD/0.5BDT/3BAPO/DCM** were selected as the most promising for optical 3D printing.

### 3.2. Influence of Resin Composition on the Resulting Polymer Antibacterial and Antifungal Activity

The antibacterial and antifungal activity of the selected vanillin acrylate-based polymer films was exhibited on a cultural medium by measuring growth inhibition zones and during the direct contact with the specimens by calculating the log reduction and percent reduction of viable microbial spores. Two reference polymer films of chitosan, a well-known antibacterial polymer and hydroxyethyl starch having no such activity were used. The dependency of the antibacterial and antifungal activity on the vanillin derivative and the presence of thiol was observed.

**Figure 10:**
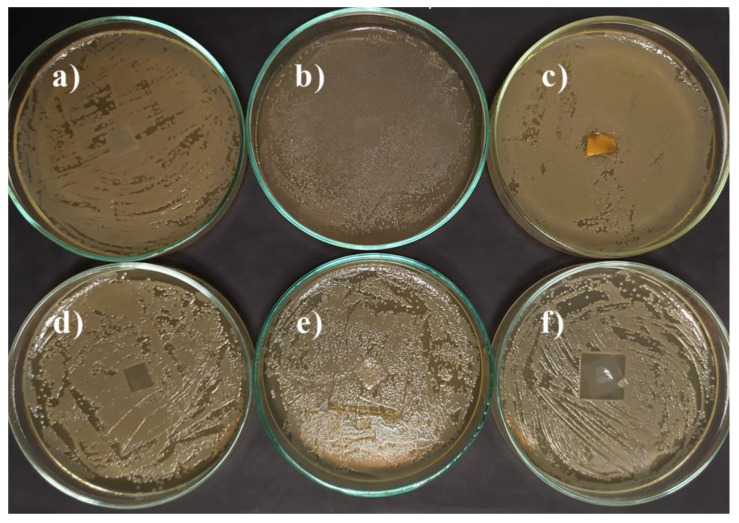
Toxicity testing of polymer film specimens for fungus *Escherichia coli* on a Mueller Hinton Agar (MHA) medium: (**a**) **1VD/1BDT/3BAPO**, (**b**) **1VD/1BDT/3BAPO/DCM**, (**c**) **1VD/3BAPO**, (**d**) **1VDM/1BDT/3BAPO/DCM**, (**e**) chitosan, (**f**) hydroxyethyl starch.

**Figure 11:**
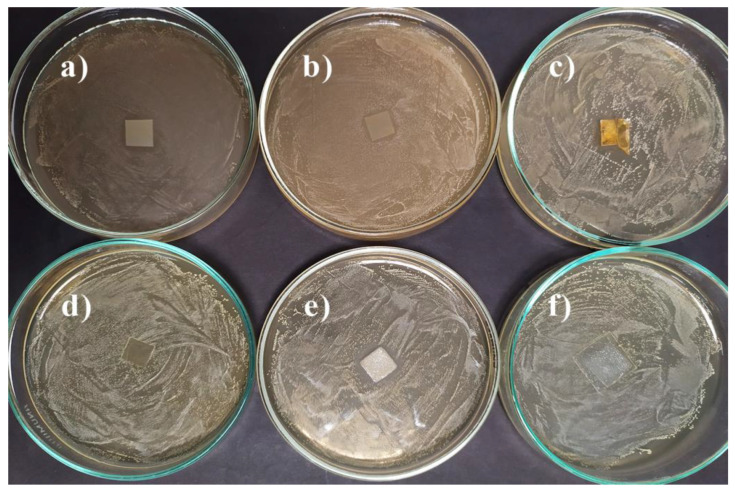
Toxicity testing of polymer film specimens for fungus *Staphylococcus aureus* on a MHA medium: (**a**) **1VD/1BDT/3BAPO**, (**b**) **1VD/1BDT/3BAPO/DCM**, (**c**) **1VD/3BAPO**, (**d**) **1VDM/1BDT/3BAPO/DCM**, (**e**) chitosan, (**f**) hydroxyethyl starch.

During the development of bacteria on the culture medium, only a slight inhibition of **1VD/3BAPO** and chitosan films by *E. coli* was observed (Table 1, Figure 10 and Figure 11.). The growth inhibition of Gram-positive bacterium *S. aureus* was slightly more pronounced. In this case, almost all the films tested were active except **1VDM/1BDT/3BAPO/DCM** and the hydroxyethyl starch film. It was determined that only the films prepared by radical photopolymerization were active against *E. coli* and only VD-based films were active against *S. aureus*. VDM-based films were less active against microorganisms probably due to the lower concentration of carbonyl groups, the presence of which has a positive effect on the antimicrobial activity of compounds [65].

The vanillin acrylate-based polymers showed a more pronounced antibacterial activity by the direct spore contact with the films (Table 1). Almost all the films tested and chitosan completely abolished the bacterial viability after 24 h of contact with spores, with the exception of **1VDM/1BDT/3BAPO/DCM** and hydroxyethyl starch films. The polymer film of **1VDM/1BDT/3BAPO/DCM** reduced the number of viable *E. coli* spores to log 1.98, and the loss of viability spores was 98.96%. Meanwhile, the hydroxyethyl starch film reduced the number of *E. coli* and *S. aureus* spores to log 1.25 and 2.85, respectively, and the loss of viability spores was 94.42 and 99.86%, respectively. It was determined that VD-based films had a better antibacterial activity than VDM-based films against *E. coli,* but all the tested polymers showed the same antibacterial activity against *S. aureus*.

**Figure 12:**
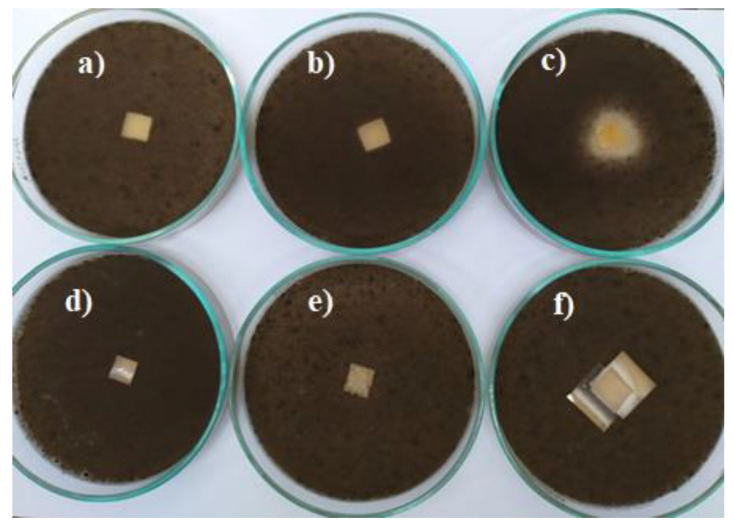
Toxicity testing of polymer film specimens for fungus *Aspergillus niger* on a Malt Extract Agar (MEA) medium: (**a**) **1VD/1BDT/3BAPO**, (**b**) **1VD/1BDT/3BAPO/DCM**, (**c**) **1VD/3BAPO**, (**d**) **1VDM/1BDT/3BAPO/DCM**, (**e**) chitosan, (**f**) hydroxyethyl starch.

**Figure 13:**
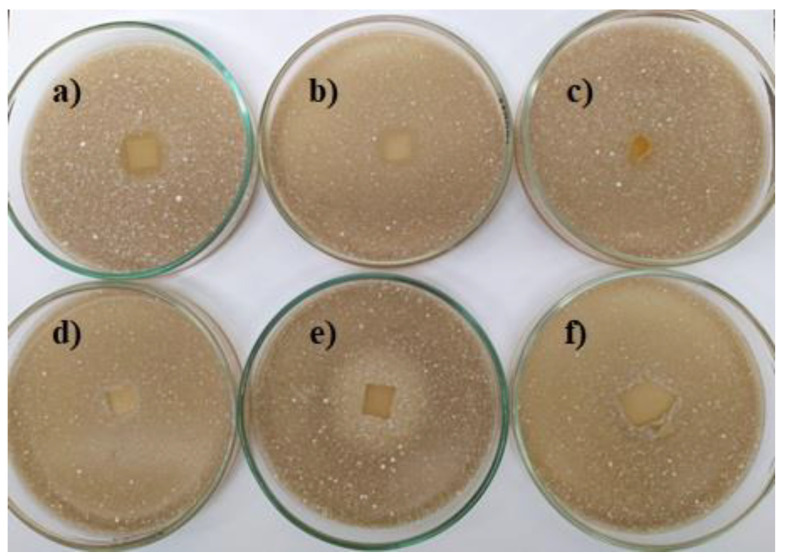
Toxicity testing of polymer film specimens for fungus *Aspergillus terreus* on a MEA medium: (**a**) **1VD/1BDT/3BAPO**, (**b**) **1VD/1BDT/3BAPO/DCM**, (**c**) **1VD/3BAPO**, (**d**) **1VDM/1BDT/3BAPO/DCM**, (**e**) chitosan, (**f**) hydroxyethyl starch.

The antifungal activity of vanillin-based polymers depended on the fungal species. The growth of *A. niger* on the culture medium was slightly inhibited only by **1VD/3BAPO**, while *A. terreus*—was inhibited by **1VD/1BDT/3BAPO** and **1VD/1BDT/3BAPO/DCM** films (Table 2, Figure 12 and Figure 13). The antifungal activity against *A. niger* was visible only for the VD-based film prepared without thiol. The addition of thiol resulted in the antifungal activity against *A. terreus.* Moreover, *A.niger* was affected by a higher concentration of carbonyl groups, which are less active against fungi than against bacteria [66], while *A. terreus* was only affected by a higher concentration of carbonyl groups in the VD-based film prepared without thiol and the presence of sulphur with the great antifungal activity [67] in VD-based films prepared with thiol. The main reason for these results is the higher concentration and the great antifungal activity of sulphur, which is present in VD-based films prepared with thiol [68].

The antifungal activity of vanillin acrylate-based polymer films was more pronounced when a direct contact of the fungal spores with the films was used, but it depended on the fungal species, as well. The polymer films of **1VD/1BDT/3BAPO**, **1VD/1BDT/3BAPO/DCM**, and chitosan completely abolished the viability of *A. niger* within 24 h due to the higher concentration of carbonyl groups and sulphur, as mentioned earlier. The viability of *A. terreus* was reduced the most by the film of **1VD/1BDT/3BAPO/DCM** with 99.98% of spores losing viability. Other VD-based polymer films pronounced a similar antifungal activity for *A. terreus.* Only the antifungal activity of **1VDM/1BDT/3BAPO/DCM** was less pronounced for this fungus as the concentration of antifungal agents was lower in the VDM-based film. The results of studies performed by different methods differ since film biocides are not released into the environment and act more efficiently in contact with the microorganism [69].

## 4. Conclusions

All components of the resins have a significant influence on photocuring kinetics and properties of the resulting polymers. The addition of solvent into the resin slowed down the photocuring process and less rigid polymers were obtained. The addition of thiol increased the photocuring rate but reduced the rigidity of the resulting polymers. Dual curing, combining free-radical and thiol-Michael mechanisms, resulted in a slightly slower photocuring process than the thiol-Michael photopolymerization, but more rigid polymers were formed. The reaction rate of the selected vanillin acrylate-based resins was similar or even faster than that of commercial petroleum-based acrylate resins for optical 3D printing. However, the resulting polymers were slightly less rigid. Vanillin acrylate- and vanillin methacrylate-based polymers showed a significant antibacterial activity against *Escherichia coli* and *Staphylococcus aureus* in direct contact and on the medium. Toxicity to the microscopic fungus *Aspergillus niger* and *Aspergillus terreus* was less pronounced, the viability of *Aspergillus niger* spores in direct contact was reduced by all the investigated vanillin acrylate-based resins. The viability of *Aspergillus terreus* was also reduced by the vanillin diacrylate-based resins, although the reduction of viability by the vanillin dimethacrylate-based polymer was lower. Vanillin diacrylate-based dual curing systems were selected as the most promising for optical 3D printing of bio-based polymers with an antibacterial and antifungal activity.

## Figures and Tables

**Table 1:** Antibacterial activity characteristics of polymer film specimens.

Polymer Film	*Escherichia coli*			*Staphylococcus aureus*			
	Growth Inhibition Zone, mm	Log Reduction after 24 h	Percent Reduction after 24 h		Growth Inhibition Zone, mm	Log Reduction after 24 h	Percent Reduction after 24 h
**1VD/1BDT/3BAPO**	0	0	100		2.1 ± 0.8	0	100
**1VD/1BDT/3BAPO/DCM**	0	0	100		2.0 ± 0.0	0	100
**1VD/3BAPO**	1.0 ± 0.7	0	100		2.1 ± 0.8	0	100
**1VDM/1BDT/3BAPO/DCM**	0	1.98	98.96		0	0	100
Chitosan	2.0 ± 0.7	0	100		2.5 ± 0.5	0	100
Hydroxyethyl starch	0	1.25	94.42		0	2.85	99.86

**Table 2:** Antifungal activity characteristics of polymer film specimens.

Polymer Film	*Aspergillus niger*			*Aspergillus terreus*		
	Growth Inhibition Zone, mm	Log Reduction after 24 h	Percent Reduction after 24 h	Growth Inhibition Zone, mm	Log Reduction after 24 h	Percent Reduction after 24 h
**1VD/1BDT/3BAPO**	0	0	100	2.2 ± 0.4	3.15	99.93
**1VD/1BDT/3BAPO/DCM**	0	0	100	2.2 ± 0.4	3.70	99.98
**1VD/3BAPO**	3.2 ± 0.8	2.33	99.54	0	3.40	99.96
**1VDM/1BDT/3BAPO/DCM**	0	2.89	99.87	0	2.92	99.88
Chitosan	0	0	100	0	2.11	99.23
Hydroxyethyl starch	0	3.07	99.92	0	2.35	99.56

## Data Availability

The data presented in this study are available on request from the corresponding author.
